# A Key Tumor Suppressor Protein Ensures Proper Chromosome Segregation during Cell Division

**DOI:** 10.1371/journal.pbio.0040065

**Published:** 2006-02-14

**Authors:** Mary Hoff

Cell division in animals is an exquisitely exact process. Before a cell splits, it has one set of chromosomes and one centrosome, a structure just outside the nucleus that plays an important part in the process. The chromosomes replicate, the centrosome duplicates, and the resulting two centrosomes migrate to opposite sides of the nucleus. Each centrosome then sends out threads that grab onto and reel in one of the two sets of chromosomes. The result: two identical “teams” that go on to make up the nuclei of the two daughter cells.

Or that's the ideal, anyway. Sometimes when cells divide they make more than two centrosomes. And sometimes cells with more than two centrosomes have daughter cells with more or fewer than the normal allotment of chromosomes. Scientists have suspected for a century that the two phenomena—“supernumerary” centrosomes and the wrong number of chromosomes, called “aneuploidy,” a common feature of cancer and premalignant cells—are causally linked. But, like the proverbial chicken and egg, it hasn't been clear which comes first. Do extra centrosomes sometimes cause aneuploidy? Or does aneuploidy create changes that make cells go overboard when duplicating centrosomes? The answer could help shed valuable light on the process that turns normal cells malignant.

Kimberly McDermott, Thea Tlsty, and colleagues explored these questions in a series of experiments reported in a new study. Using laboratory techniques that allowed them to observe the number and distribution of chromosomes and centrosomes at various stages of cell division, the researchers showed that the production of supernumerary centrosomes can in some cases lead to aneuploidy. They also unraveled the process by which supernumerary centrosomes develop, and showed that when supernumerary centrosomes are present, cell division produces lopsided teams of chromosomes that aren't able to play by the rules of normal cells.

To accomplish all this, the researchers focused their attention on human mammary epithelial cells (HMECs). Most HMECs can only be grown in culture for a limited amount of time, after which they stop dividing; however, a subpopulation of these cells, called vHMECs (for “variant” HMECs), can divide for longer—but eventually end up with chromosome abnormalities, including aneuploidy. The researchers discovered that if they temporarily paused chromosome replication with a reversible chemical treatment in vHMECs, some cells ended up with extra centrosomes—as though the original two centrosomes had not gotten the message that things were on hold, and duplicated again while the chromosomes sat. Knowing from other studies that a protein called p16INK4a (which normally plays a role in suppressing tumor formation) is silenced in vHMECs, they tested its possible role in the generation of extra centrosomes by blocking its expression in HMECs. As they predicted, some of the HMECs in which p16INK4a was blocked developed supernumerary centrosomes. And when they altered vHMECs to make them express p16INK4a, the tendency to produce supernumerary centrosomes was reduced.

How does the absence of p16INK4a lead to too many centrosomes? A normal centrosome contains two assemblages of microtubules, called a centriole pair, that split up and make new centriole partners when a cell divides. Looking closely at the supernumerary centrosomes, the researchers discovered a significant portion with only one centriole. This, along with the fact that cells rarely end up with more than four centrosomes, led them to speculate that p16INK4a acts as a sort of centriole pair “policeman”—when it's around, the centriole pair splits once as it is supposed to, but if it's disabled, the centriole pairs can split more than once to generate supernumerary centrosomes.

To clearly link production of supernumerary centrosomes resulting from loss of p16INK4a activity to aneuploidy, the researchers looked at cell division in vHMECs and in HMECs with and without blocked p16INK4a. They found that pausing replication was associated with increases in supernumerary centrosomes and aneuploidy in vHMECs and in HMECs with blocked p16INK4a, but not in normal HMECs. To prove a causal connection, they observed cell division using time-lapse microscopy. They found that vHMECs with more than two centrosomes were more likely to produce aneuploid daughter cells than were vHMECs with two centrosomes.

The researchers tested other cell types and discovered that loss of p16INK4a activity produces supernumerary centrosomes and aneuploidy in them as well. And, using human fibroblasts, they showed that p16INK4a prevents inappropriate centriole pair splitting by working with a gene called *p21* that inhibits the activity of Cdk, a type of protein needed for centrosome duplication.

Cells with inactive p16INK4a are found in normal human breast tissue. This study suggests that if DNA replication gets held up—which it can if DNA needs repair due to exposure to chemotherapy—such cells would produce supernumerary centrosomes. These supernumerary centrosomes could lead to aneuploidy, loss or gain of genes regulating cell division and death, and may result in the beginning stages of a tumor.

## 

**Figure:**
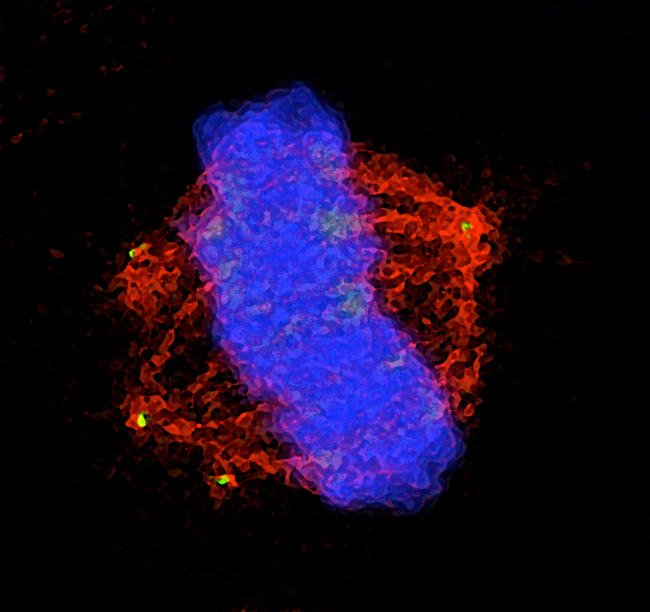
Loss of p16^INK4a^ allows cells to produce too many centrosomes (the green dots) which in turn leads to genomic instability.

